# Does the impact of case management vary in different subgroups of multimorbidity? Secondary analysis of a quasi-experiment

**DOI:** 10.1186/s12913-017-2475-x

**Published:** 2017-08-03

**Authors:** Jonathan Stokes, Søren Rud Kristensen, Kath Checkland, Sudeh Cheraghi-Sohi, Peter Bower

**Affiliations:** 1 NIHR Greater Manchester Primary Care Patient Safety Translational Research Centre, Manchester Academic Health Science Centre, University of Manchester, Manchester, UK; 2 Manchester Centre for Health Economics, Manchester Academic Health Science Centre, School of Health Sciences, University of Manchester, Manchester, UK; 3 NIHR School for Primary Care Research, Centre for Primary Care, Manchester Academic Health Science Centre, University of Manchester, Manchester, UK

**Keywords:** Multimorbidity, Case management, Integrated care

## Abstract

**Background:**

Health systems must transition from catering primarily to acute conditions, to meet the increasing burden of chronic disease and multimorbidity. Case management is a popular method of integrating care, seeking to accomplish this goal. However, the intervention has shown limited effectiveness. We explore whether the effects of case management vary in patients with different types of multimorbidity.

**Methods:**

We extended a previously published quasi-experiment (difference-in-differences analysis) with 2049 propensity matched case management intervention patients, adding an additional interaction term to determine subgroup effects (difference-in-difference-in-differences) by different conceptualisations of multimorbidity: 1) Mental-physical comorbidity versus others; 2) 3+ chronic conditions versus <3; 3) Discordant versus concordant conditions; 4) Cardiovascular/metabolic cluster conditions only versus others; 5) Mental health-associated cluster conditions only versus others; 6) Musculoskeletal disorder cluster conditions only versus others 7) Charlson index >5 versus others. Outcome measures included a variety of secondary care utilisation and cost measures.

**Results:**

The majority of conceptualisations suggested little to no difference in effect between subgroups. Where results were significant, the vast majority of effect sizes identified in either direction were very small. The trend across the majority of the results appeared to show very slight increases of admissions with treatment for the most complex patients (highest risk). The exceptions to this, patients with a Charlson index >5 may benefit slightly more from case management with decreased ACSC admissions (effect size (ES): −0.06) and inpatient re-admissions (30 days, ES: −0.05), and patients with only cardiovascular/metabolic cluster conditions may benefit slightly more with decreased inpatient non-elective admissions (ES: −0.12).

Only the three significant estimates for the musculoskeletal disorder cluster met the minimum requirement for at least a ‘small’ effect. Two of these estimates in particular were very large. This cluster represented only 0.5% of the total patients analysed, however, so is hugely vulnerable to the effects of outliers, and makes us very cautious of interpreting these as ‘real’ effects.

**Conclusions:**

Our results indicate no appropriate multimorbidity subgroup at which to target the case management intervention in terms of secondary care utilisation/cost outcomes. The most complex, highest risk patients may legitimately require hospitalisation, and the intensified management may better identify these unmet needs. End of life patients (e.g. Charlson index >5)/those with only conditions particularly amenable to primary care management (e.g. cardiovascular/metabolic cluster conditions) may benefit very slightly more than others.

**Electronic supplementary material:**

The online version of this article (doi:10.1186/s12913-017-2475-x) contains supplementary material, which is available to authorized users.

## Background

It is widely agreed that health systems must transition from catering primarily to acute conditions, to meet the increasing burden of chronic disease and multimorbidity. New care models have been called for to achieve this goal, based in primary care and ‘integrated’ with wider health and care sectors [1]. In practice, these new interventions and models are based primarily around the concept of ‘case management’ [2, 3]. Case management involves case finding (identifying ‘high-risk’ individuals to case manage), individual assessment, care planning and care co-ordination (with regular review, monitoring and adaptation of the care plan) [4]. Many use a multidisciplinary team (MDT) to case manage, which may combine ‘professional integration’ with co-ordination activity [5].

The logic of the case management model rests on the presence of so-called ‘super-utilizers’ [6]. This is a small group of ‘high-risk’ patients (almost exclusively multimorbid [7]) that utilize a disproportionate amount of healthcare resource. For example, the United States Government Accountability Office estimate that the most expensive 5% of Medicaid-only enrolees accounted for 48% of Medicaid-only health spending each year from 2009 to 2011 [8]. The assumption of case management is that by targeting additional and individually tailored primary care at these patients, more costly secondary care admissions (particularly emergency admissions) can be avoided. Thus, overall healthcare spending costs can be reduced, patients can be treated in an environment more satisfactory to them, and in a more holistic and preventative way [9].

However, research suggests that case management does not meet its primary aims for those directly managed, although patient satisfaction does appear to be increased [10, 11].

In practice, MDT case management tends to target those identified as ‘high risk’ using a selected statistical algorithm that is validated to predict those patients likely to have substantial future healthcare use. These tools generate a heterogeneous group of patients, and it may be that there are subgroups for which the direct effects of the intervention are more effective. There are a number of ways of conceptualising multimorbidity, and little evidence as to the advantages and disadvantages of each [12, 13].

These conceptualisations can broadly be categorised into four distinct groups [13]. Within each of these groups, we outline the specific measures we focus on in our analysis with justification for doing so.

1. Simply counting the number of chronic conditions/medications from a pre-specified list.
  - **Count of diseases –** A simple count of conditions is the most basic conceptualisation of multimorbidity, and the most ubiquitous multimorbidity measure in the current literature [14].
2. Grouping chronic diseases by dyads or triads (i.e. clustering).
  - **Disease clusters –** As the search for more clinically meaningful conceptualisations of multimorbidity advances, identification of ‘non-random clustering’ of diseases (i.e. associative multimorbidity) has been a common theme in the literature. A number of plausible mechanisms can explain disease clustering, as outlined by Valderas et al. [15]. Furthermore, identifying commonly co-occurring sets of diseases may have important implications for developing clinical guidelines for patients with multiple chronic conditions, for which the current single-disease approach can cause issues [16].
3. Using an index of variable complexity.
  - **Charlson index –** A number of indices of multimorbidity have emerged, to attempt to better account for the variation in severity of different diseases. No index is more established and prevalent in the literature than the Charlson index, which weights (giving a score between 1 and 6) relevant diseases, and gives a summed score of the weights to the individual [14].
4. Identifying homogeneous groups of people with common diseases and characteristics.
  - **Mental-physical comorbidities –** These patients are at increased risk for active and precursors to patient safety incidents in primary care [17]. Moreover, a common mental health condition, depression, has been shown to be particularly important in modifying multimorbidity management and outcomes [18].
  - **Discordant comorbidities –** i.e. co-occurring diseases that are not managed synergistically. Discordant conditions are likely to add to the complexity of clinical treatment/decision-making [19], potentially putting this group at increased risk of management failures. This is opposed to concordant conditions (i.e. co-occurring diseases that are managed synergistically).

To explore whether the effects of case management vary in patients with different types of multimorbidity, we extend an existing analysis [20] using these different definitions of multimorbidity to stratify patients within the sample.

## Methods

Detailed methods for the analysis models can be found in our previous publication and the accompanying appendix (see individual-level direct effects model/high-risk subgroup effects model) [20].

Briefly, we evaluated effectiveness of a MDT case management intervention (practice integrated care teams – PICT) in a single Clinical Commissioning Group (CCG) in the UK NHS. Patients were selected for the intervention primarily based on a predictive risk model (the Combined Predictive Risk Model), using previous inpatient utilisation data to calculate a risk score of a patient’s likelihood of re-hospitalisation within the next year [21]. As an evolving complex intervention, however, in practice there was also qualitative evidence that some patients were included in the intervention based on clinical judgement (with some clinicians not favouring use of the risk model) [22].

We matched 2049 intervention patients (identified in the data by the CCG) one-to-one with controls from the same CCG using propensity scores to ensure the intervention and control groups were comparable in terms of risk for the statistical analysis (regardless of how they were recruited). Patients were matched based on the observable characteristics for which the patients were recruited in practice to maximise comparability (based on age; sex; index of multiple deprivation (IMD) 2010; total multimorbidity count previous to the first available start date of our intervention patients; previous inpatient, outpatient and A&E attendance in the previous year before the first intervention patient start date) [21]. Intervention participants and controls were well matched based on pre-intervention observables (see Additional file 1).

We analysed anonymised data held by the CCG relating to hospital admissions and costs. Outcome measures were summed to a count per patient per month over the period September 2010 to March 2015 inclusive, allowing a three-year pre-intervention trend period (patients joined the intervention gradually from September 2013 to February 2015). Primary outcome measures included A&E visits; inpatient non-elective stays, 30-day re-admissions; inpatient elective stays; outpatient visits; and admissions for ambulatory care sensitive conditions. Secondary measures included: inpatient length of stay; total cost of secondary care services.

Previously, we used difference-in-differences (DD) analysis. This method is a quasi-experiment, where the intervention group is compared to a control group (constructed retrospectively, and known to be unaffected by the intervention). The method compares the difference in a measured outcome between intervention and control groups in a pre-intervention period (difference 1), and compares to the difference between the two groups after an intervention is introduced (difference 2), attributing this second difference to the intervention effect. We used a time fixed effect instead of the usual binary post dummy to account for the gradual intervention joining, comparing appropriately at each time point [23, 24].

In this study, we use difference-in-difference-in-differences (DDD) analysis, which adds an additional interaction term to a standard DD approach used in the original study. This allows us to observe subgroup effects (of the different multimorbidity conceptualisations) of the intervention, i.e. the effects of the intervention on a subgroup over and above any baseline DD effect. The models were negative binomial count models (except for total cost of secondary care which was better represented by a zero-inflated negative binomial model based on admission events). We adjusted for age, index of multiple deprivation (IMD) domains (excluding health), practice- and time-fixed effects. We clustered our standard errors by practice to deal with concerns of serial correlation [25]. We report the effect size (standardised mean difference) as a measure of practical significance, calculated from the average partial effect (reported in the Additional file 1). All data preparation and analysis was carried out using STATA (version 13) [26].

From a list of 20 chronic conditions (see Additional file 1) represented in the NHS quality and outcomes framework (QOF), we created a number of dummy variables representing different conceptualisations of multimorbidity:

- Count of diseases - a simple cumulative count of the individual’s conditions. We created a dummy for 3+ (thought to be a more discriminating definition of multimorbidity than 2+ conditions, better identifying patients with higher needs [27]) versus <3 conditions.
- Disease clusters – We stratified by three common disease clusters identified in the literature [16]. We created a dummy comparing those that only have one or more of the diseases in that cluster versus the rest of the patients in the analysis.
  - Cardiovascular/metabolic cluster: Diabetes, Hypertension, Chronic Heart Disease, Obesity
  - Mental health-associated cluster: Mental health condition (Schizophrenia/Bipolar Disorder/Psychoses/Depression), Hypothyroidism, Dementia, Asthma, Chronic Obstructive Pulmonary Disease (COPD), Rheumatoid Arthritis, Obesity
  - Musculoskeletal disorder cluster: Rheumatoid Arthritis, Osteoporosis, Obesity
- Charlson index - an established measure, with its own set of relevant chronic conditions and weightings, we used the STATA command ‘*charlson*’ to record a Charlson index for each participant [28]. We created a dummy comparing those with a Charlson index >5 (suggested in the literature to be those patients at ‘highest risk’ of negative outcomes [29]) to all other patients.
- Mental-physical comorbidities – There were a number of mental health conditions in our disease list (depression, schizophrenia, bipolar disorder, psychoses, and dementia). We created a dummy variable comparing those patients with both a mental and physical conditions to all other patients.
- Discordant comorbidities - we used a list of determined concordant conditions that share a vascular aetiology and common chronic management and treatment goals (coronary heart disease, chronic kidney disease, diabetes, hypertension, heart failure, stroke/transient ischaemic attack, atrial fibrillation, and peripheral vascular disease), and classified the remainder as discordant conditions [30]. We created a dummy variable comparing patients with discordant conditions to all other patients.

With multiple comparisons, the risk of type I errors is inflated [31]. As a sensitivity analysis, we subsequently correct the results for multiple testing using the Holm-Bonferoni adjustment for multiple comparisons to identify potential false positive results [32]. We report those results that remained significant with the adjusted threshold at the end of the results section.

## Results

Each of the dummy variables selected a different proportion of the total patient group analysed (*n* = 4098), as complex/‘higher risk’ (see Table 1). The proportion of patients selected in total varied by measure. The count (3 or more chronic diseases compared to all others), and discordant comorbidities dummies were least selective, including over half of the total patient group in each case. The specific disease clusters were, as expected, the most selective. The musculoskeletal disorder cluster (rheumatoid arthritis, osteoporosis and obesity), for example, selected only 0.5% of the total patient group in the positive dummy group.

**Table 1:** Number (and proportion) of patients positively identified by each dummy variable

	Number of patients positively identified in that dummy measure		
Multimorbidity measure	PICT (*n* = 2049)	Controls (*n* = 2049)	Total (*n* = 4098)
Mental/physical condition	319 (15.6%)	498 (24.3%)	817 (19.9%)
3 + conditions	992 (48.4%)	1165 (56.9%)	2157 (52.6%)
Discordant comorbidities	1030 (50.2%)	1208 (59.0%)	2238 (54.6%)
Cardiovascular/metabolic cluster	300 (14.6%)	209 (10.2%)	509 (12.4%)
Mental health-associated cluster	187 (9.1%)	234 (11.4%)	421 (10.3%)
Musculoskeletal disorder cluster	12 (0.6%)	8 (0.4%)	20 (0.5%)
Charlson >5	398 (19.4%)	347 (16.9%)	745 (18.2%)

PICT = Practice integrated care teams (intervention)

Table 2 shows the correlation between each of the dummy variables used to select patients. The less selective dummy variables (mental/physical conditions; 3 or more conditions; and discordant comorbidities) were well correlated with each other. The remaining more selective measures (the three literature clusters; Charlson index) were far less correlated with any other.

**Table 2:** Correlation between multimorbidity dummies used, describing the overlap of patients selected in each grouping, where 1 implies a perfect overlap (i.e. all patients identified positively by both multimorbidity conceptualisations being compared) and lower values imply less overlap (i.e. fewer of the same patients identified positively by both multimorbidity conceptualisations being compared)

Multimorbidity Conceptualisation	Mental/physical condition	3 + conditions	Discordant comorbidities	Cardiovascular/metabolic cluster	Mental health-associated cluster	Musculoskeletal disorder cluster	Charlson >5
Mental/physical condition	1.00						
3 + conditions	0.32	1.00					
Discordant comorbidities	0.45	0.65	1.00				
Cardiovascular/metabolic cluster	−0.19	−0.28	−0.37	1.00			
Mental health-associated cluster	−0.01	−0.30	−0.16	−0.10	1.00		
Musculoskeletal disorder cluster	−0.03	−0.07	−0.08	0.08	0.16	1.00	
Charlson >5	−0.02	0.01	−0.02	−0.17	−0.16	−0.03	1.00

**Fig. 1:**
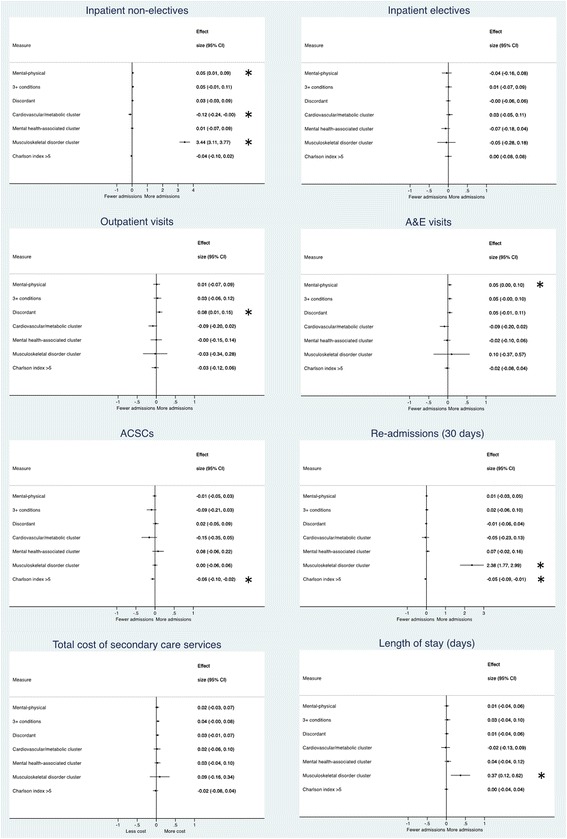
Forest plot comparing multimorbidity measures by each outcome. Effect size = standardised mean difference. * = statistically significant result (*p* < 0.05). ACSCs = admissions for ambulatory care sensitive conditions

Figure 1 shows a forest plot for each outcome measure, comparing the multimorbidity subgroups within each. The effect sizes represent the impact of the treatment for the group identified as highest risk (i.e. those positively identified as multimorbid according to that conceptualisation) within each dummy. Estimates which lie to the left of the line of no effect (0) favour the intervention for that multimorbid-defined group (i.e. indicate decreased utilisation/cost). Estimates that lie to the right of the line show that intervention treatment led to increased utilisation/cost for that group and outcome measure. Only estimates whose 95% confidence interval does not cross the line of no effect are statistically significant. The table in the Additional file 1 gives the full list of regression results (i.e. adjusted intervention effect as the difference per patient per month) that these effect sizes were calculated from.

The results from Fig. 1 indicate that for the majority of outcomes, the different conceptualisations of multimorbidity have given broadly the same interpretation. For inpatient electives, and total cost of secondary care outcomes, none of the conceptualisations held a statistically significant difference. For outpatient visits (discordant conditions; effect size (ES): 0.08), A&E visits (mental-physical comorbidities; ES: 0.05), and ACSCs (Charlson index >5; ES: -0.06), there was a single (but different) multimorbidity concept which differed significantly. However, all of these differences were to a very small extent (effect size less than the threshold for a ‘small’ effect, 0.2, in all cases [33]).

Most notably, the musculoskeletal disorder cluster deviated significantly from the other results across a number of outcomes (inpatient non-electives, ES: 3.44; 30-day re-admissions, ES: 2.38; and length of stay, ES: 0.37). In each of these measures, the results suggested that the intervention increased the utilisation of inpatient services for patients with these conditions, with large effect sizes (particularly for inpatient non-electives and 30-day re-admissions).

The significant results for the other multimorbidity measures were far more conservative, but tended to follow this similar trend, suggesting that the highest risk patients increased utilisation following treatment. The exception to this was for the Charlson index, where the effect of case management on those with Charlson >5 led to a larger decrease than those with Charlson <5 for ambulatory care sensitive conditions (ACSCs – effect size (ES): −0.06) and 30-day inpatient re-admissions (ES: −0.05). Those patients with only conditions from the cardiovascular/metabolic cluster also appeared to have slightly fewer inpatient non-elective admissions following treatment (ES: −0.12).

After Holm-Bonferroni correction was applied to all results, only two of the statistically significant results held: the findings of significant increases following treatment of inpatient non-elective admissions and 30-day re-admissions for patients with musculoskeletal disorder cluster conditions.

## Discussion

### Summary of findings

#### Do different operationalisations of multimorbidity give different answers in terms of MDT case management effectiveness, and if so, why?

As outlined above, the majority of conceptualisations gave very similar results in relation to effectiveness of the intervention, suggesting little to no difference in effect between subgroups. Where results were significant, the vast majority of effect sizes identified in either direction were very small. Cohen’s rule of thumb for interpreting effect sizes is that 0.2 indicates a small effect, 0.5 a medium, and 0.8 a large effect [33]. Only the three significant estimates for the musculoskeletal disorder cluster (rheumatoid arthritis, osteoporosis and obesity) met the minimum requirement for at least a ‘small’ effect. Two of these estimates in particular were very large (inpatient non-elective admissions ES: 3.44; and 30-day re-admissions ES: 2.38). Musculoskeletal disorders are associated with some of the poorest quality of life, particularly because of the bodily pain and poorer level of physical functioning associated with them [34]. These conditions are difficult to manage leading to a large economic and social burden (with a large proportion of the cost burden due to hospital inpatient admissions) [35]. Perhaps, this difficulty in managing these complex conditions is not alleviated by the MDT, but simply draws attention to unmet need requiring escalation to emergency services? However, interpretation of these large effect sizes in light of the tiny numbers of patients in this group (only 0.5% of the total population analysed) makes us extremely cautious of interpreting these as ‘real’ effects. These small numbers make this subgroup result hugely vulnerable to the effects of outliers.

#### What do these results imply for the intervention in practice?

The trend across the majority of the results appeared to show very slight increases of admissions with treatment for the most complex patients (highest risk), again, perhaps indicating identification of unmet need which may plausibly result from an intervention of this type. This would also agree with the sub-group finding in our original analysis paper, where we stratified by risk-tool score, finding that the highest risk patients treated appeared to benefit least [20].

However, an important exception to this finding was for those patients with a Charlson index >5, where those patients may benefit from slightly decreased ACSC admissions (ES: −0.06) and 30-day inpatient re-admissions (ES: −0.05) with the intervention. The Charlson index, unlike the other measures, was developed primarily as a method of predicting mortality [36]. Those with an index greater than or equal to 5 have an 85% chance of 1-year mortality [29]. Perhaps these generally ‘end of life’ patients are managed differently by the case management team than others? For instance, the treatment plan may be more focused on decreasing the burden of care (e.g. medications – perhaps being less likely to have adverse reactions) at the end of life, or attention may shift to palliative care closer to home resulting in different secondary care use?

Those with conditions only from the cardiovascular/metabolic cluster (diabetes, hypertension, chronic heart disease, obesity), may also have benefited from slightly decreased inpatient non-elective admissions (ES: −0.12). These conditions should all be manageable in primary care [37], perhaps explaining that hospital admissions for these patients may be most susceptible to decrease with increased primary care. However, general ACSC admissions were not significantly affected for this patient group (although the trend was in the same direction; ES: −0.15).

### Strengths and weaknesses

As with any subgroup analysis, power to detect subgroup effects will suffer proportionately more than power to detect the overall effect [38]. Therefore, we present this as a very preliminary analysis, results of which may be added to by future studies using similar conceptualisations of multimorbidity. In addition, the interpretation of the subgroup effects should be made in light of the overall effect. These represent the differential effects of the intervention in terms of that subgroup, and may be in the context of an overall null effect, for example.

The strengths and weaknesses identified in our primary analysis publication similarly apply to this analysis [20]. Particularly relevant to this analysis, we previously identified potential spill-over effects of the intervention at the practice-level. If spill-over effects did indeed affect other patients in the practice, then the individual-level effects may be driven towards the null (as the control group was also sampled from implementing practices). This is similarly true for the DDD analysis conducted here. However, these spill-over effects were not strongly indicated at the practice level. Furthermore, if our hypothesis is that these practice-level spill-over effects are as a result of preventative MDT working benefiting lower risk patients predominantly, then the spill-over may not apply to the high-risk matched group analysed in this sample at all. Also important to note, the outpatient visits outcome failed the parallel pre-trends test, and this outcome may therefore be biased in favour of the intervention group.

Although we would have preferred to use chronic conditions data recorded in primary care for our multimorbidity measure, this data was unavailable for this study. However, as high-risk patients (both the intervention and propensity matched controls), these are probably the most likely to encounter inpatient admissions (and indeed were selected for ‘high risk’ of admissions). Therefore, these patients should in theory have the most complete recordings at this service level in comparison to the general population. From previous literature though, we can expect our multimorbidity measure to be less sensitive (i.e. be a predictably lower count) because it comes purely from hospitalisation data [39]. Nevertheless, our multimorbidity measure gained strength from being based on a list of 20 chronic conditions, deemed particularly important in the UK’s NHS setting, which follows guidance from the multimorbidity literature [27].

Using only secondary care data gives us a somewhat limited indication of the intervention on overall system costs. However, the intervention logic model hypothesises that there will be a substitution effect of replacing more costly secondary care with less costly primary care, so following this model, secondary care costs should give a good proxy for effects on these overall system costs. Nevertheless, using linked health and care data (including not only secondary care, but also primary, tertiary and social care) might allow effects of similar interventions on overall system spending to be better evaluated and managed.

### Results in relation to other studies

There is little secondary analysis data available on multimorbidity from studies to date [40]. The authors are unaware of any multimorbidity subgroup analysis from evaluations of case management interventions to compare results directly. With the limited power of a single subgroup analysis, there is therefore the need for others to report these type of results, allowing future meta-analysis (or individual patient data meta-analysis, which may be a useful model to adopt [41]) and confirming of initial findings. The single comparison we can make presently is to our original publication, where we stratified results by risk-tool score [20]. As discussed above, the majority of the results obtained here confirmed our findings from the original subgroup analysis, i.e. that generally higher risk, more complex patients, tended towards increased secondary care utilisation with treatment, perhaps indicating identification of unmet need. The exceptions were from our stratification by Charlson index and the cardiovascular/metabolic cluster, which may have been identifying slightly different and distinct populations (those approaching the end of life with the Charlson index, and those with conditions most amenable to primary care with the cardiovascular/metabolic cluster). This potentially explains the bucking of this trend in these patient groups.

### Implications for clinicians and policymakers

Our analysis suggests that there is little to differentiate the effectiveness of the case management intervention by targeting specific multimorbid groups, regardless of conceptualisation. Where we identified subgroups where the intervention may be more beneficial (Charlson index >5 & cardiovascular/metabolic cluster), the differences were extremely small. We hypothesise that these differences may be due to differences in treatment at the end of life/treatment of only conditions that are particularly manageable in primary care. In the majority of cases, however, we expect the intervention to uncover unmet need, particularly for the most complex (highest risk) patients. As stated above, these are currently preliminary findings, and await further testing on other datasets. For example, other case management interventions using alternative participant selection criteria may select participants more amenable to the intervention (so-called ‘impactibility’) [42], potentially altering the results overall, and maybe also for selected multimorbid subgroups.

### Future research

Managing overall healthcare spending and over-utilisation of secondary care remains a vital goal in health systems globally. Further research is needed to evaluate how we may accomplish this goal, particularly for the ever-increasing numbers of multimorbid patients who are inadequately managed and at a high price in our current systems. Case management does not appear to be the singular solution.

These results do not suggest that any of the different ways of conceptualising multimorbidity perform better than any others in understanding the impact of case management. However, in assessing interventions, secondary analyses by multimorbidity subgroup may be a valuable tool to identify specific targetable groups [40], to achieve maximum cost-effectiveness from an intervention. As we illustrate here, there are numerous ways to operationalise multimorbidity, and perhaps utilising a range of these may hint at subtler indications of intervention effectiveness (or ineffectiveness) which may be explored through yet further analysis (e.g. qualitative methods to explore potential mechanisms of action). When doing so, it is important to adjust for multiple testing in sensitivity analysis, to avoid drawing overly strong conclusions which may be based on false positive results.

Alternatively, other factors than multimorbidity may be of relevance – such as social care needs, or frailty.

## Conclusions

Our results indicate no appropriate multimorbidity subgroup at which to target the MDT case management intervention in terms of secondary care utilisation/cost outcomes. The most complex, highest risk patients may legitimately require hospitalisation, and the intensified management may better identify these unmet needs. However, end of life patients/those with only conditions particularly amenable to primary care management may benefit a very small amount more than others. There is an on-going need to find appropriate ways of addressing health system spending and management of multimorbid patients, however the concept is defined [43].

## Additional file

Additional file 1: Intervention to control matching, Multimorbidity measures, Results table. Table comparing intervention and control characteristics before and after matching. List of conditions and ICD-10 codes used to construct the multimorbidity measures. Detailed regression results table, the basis for results shown in Fig. 1 in the main manuscript. (PDF 644 kb)

## Abbreviations

A&E: Accident & emergency
ACSCs: admissions for ambulatory care sensitive conditions
CCG: Clinical Commissioning Group
COPD: Chronic Obstructive Pulmonary Disease
DD: Difference-in-differences
DDD: Difference-in-difference-in-differences
IMD: Index of multiple deprivation
LOS: Length of stay
MDT: Multidisciplinary team
PICT: Practice integrated care teams
QOF: Quality and outcomes framework
