# Supplementary material for: Does the impact of case management vary in different subgroups of multimorbidity? Secondary analysis of a quasi-experiment
Source: BMC Health Serv Res. 2017 Aug 3;17:521. doi: 10.1186/s12913-017-2475-x (PMC5543754; doi:10.1186/s12913-017-2475-x)
Supplement: Additional file 1: — Intervention to control matching, Multimorbidity measures, Results table. Table comparing intervention and control characteristics before and after matching. List of conditions and ICD-10 codes used to construct the multimorbidity measures. Detailed regression results table, the basis for results shown in Fig. 1 in the main manuscript. (PDF 644 kb) [file 12913_2017_2475_MOESM1_ESM.pdf]

SUPPLEMENTARY FILE

INTERVENTION PARTICIPANT TO CONTROL MATCHING

Individual baseline characteristics (before and after matching)

	Before matching			After matching		
Mean (unless otherwise indicated)	PICT (SD)	Controls (SD)	SMD	PICT (SD)	Controls (SD)	SMD
N	2049	93 532		2049	2049	
Male (%)	44.3	47.4		44.3	44.1	
Age	67.2 (17.8)	35.3 (22.2)	−1.44	67.2 (17.8)	65.8 (18.7)	−0.07
IMD 2010	40.2 (14.8)	40.6 (16.0)	0.03	40.2 (14.8)	40.2 (15.8)	0.00
MM count baseline	2.7 (2.1)	0.7 (1.2)	−1.63	2.7 (2.1)	2.4 (2.2)	−0.12
Previous inpatient admissions	1.3 (2.1)	0.3 (1.1)	−0.88	1.3 (2.1)	1.2 (2.2)	−0.05
Previous outpatient visits	7.0 (9.6)	1.9 (4.3)	−1.14	7.0 (9.6)	7.2 (9.8)	0.02
Previous A&E visits	1.4 (2.4)	0.5 (1.2)	−0.73	1.4 (2.4)	1.4 (2.5)	0.00

- Previous admissions calculated for period 31 August 2012–1 September 2013 (12 months prior to the first intervention patient start date).
- A&E, accident and emergency; IMD, Index of Multiple Deprivation; PICT, Practice Integrated Care Team; SMD, standardised mean difference.

MULTIMORBIDITY MEASURES

The multimorbidity measures (except Charlson index which has its own pre-specified list of condition codes [1]) were prepared from the previously recorded (period June 2006 to March 2015) inpatient admissions, for 20 chronic conditions recorded in the NHS Quality and Outcomes Framework (QOF) (see below for list of conditions and icd-10 codes) [2-4].

1. Asthma

J45-J47

2. Atrial fibrillation

I48

3. Cancer

C00–C14, C15–C26, C30–C39,

C40–C41, C43–C44, C45–C49,

C50, C51–C58, C60–C63,

C64–C68, C69–C72, C73–C75,

C81–C96, C76–C80, C97,

D00–D09, D37–D48 (Koller et al 2014)

4. Chronic kidney disease

N18

5. Coronary heart disease

I20-I25

6. COPD

J40-J44

7. Dementia

F00-F03

8. Depression

F32-F33

9. Diabetes mellitus

E10-E14

10. Epilepsy

G40-G41

11. Heart failure

I50

12. Hypertension

I10-I15

13. Hypothyroidism

E00-E03

14. Learning disability

F80-F89

15. Mental health (schizophrenia, bipolar affective disorder and other psychoses)

F20-F29, F31, F34-F39

16. Obesity

E66

17. Osteoporosis

M80-M82

18. Atherosclerosis/Peripheral arterial oclusive disease (PAOD)

I65–I66, I67.2, I70, I73.9

19. Rheumatoid arthritis

M05-M06

20. Cerebral ischemia/chronic stroke

I60–I64, I69, G45

RESULTS TABLE

**Outcome**	**Mental-physical**	**3 or more conditions**	**Discordant versus other**
	**Count (nbreg) model. Adjusted+ intervention effect (95% CI) (difference per patient per month)**		
**Primary outcomes:**			
Inpatient non-electives	**0.0122 (0.0018 to 0.0228)#**	0.0129 (-0.0027 to 0.0286)	0.0078 (-0.0075 to 0.0232)
Inpatient electives	-0.0109 (-0.0409 to 0.0191)	0.0023 (-0.0166 to 0.0212)	-0.0008 (-0.0156 to 0.0139)
Outpatient visits	0.0169 (-0.0820 to 0.1158)	0.0393 (-0.0717 to 0.1503)	**0.0900 (0.0040 to 0.1760)#**
A&E visits	**0.0200 (0.0009 to 0.0391)#**	0.0186 (-0.0031 to 0.0402)	0.0187 (-0.0058 to 0.0432)
ACSCs	-0.0006 (-0.0050 to 0.0038)	-0.0081 (-0.0199 to 0.0036)	0.0019 (-0.0046 to 0.0084)
Re-admissions (30 days)	0.0006 (-0.0042 to 0.0053)	0.0024 (-0.0065 to 0.0113)	-0.0011 (-0.0074 to 0.0053)
**Secondary outcomes:**			
Total cost of 2o care services (£)~	13.8866 (-35.6720 to 63.4452)	36.1449 (-2.2763 to 74.5661)	24.9248 (-9.3942 to 59.2437)
Length of stay (days)	0.0612 (-0.2395 to 0.3619)	0.1814 (-0.1933 to 0.5560)	0.0496 (-0.2348 to 0.3340)

**Outcome**	**Lit Cluster 1**	**Lit Cluster 2**	**Lit Cluster 3**
	**Count (nbreg) model. Adjusted[+] intervention effect (95% CI) (difference per patient per month)**		
**Primary outcomes:**			
Inpatient non-electives	**-0.0284 (-0.0565 to -0.0003)#**	0.0014 (-0.0190 to 0.0219)	**0.8171 (0.7376 to 0.8966)#**
Inpatient electives	0.0071 (-0.0121 to 0.0263)	-0.0162 (-0.0431 to 0.0106)	-0.0109 (-0.0670 to 0.0452)
Outpatient visits	-0.1067 (-0.2334 to 0.0200)	-0.0043 (-0.1715 to 0.1628)	-0.0397 (-0.3970 to 0.3175)
A&E visits	-0.0353 (-0.0796 to 0.0090)	-0.0092 (-0.0431 to 0.0248)	0.0387 (-0.1392 to 0.2166)
ACSCs	-0.0138 (-0.0317 to 0.0042)	0.0075 (-0.0060 to 0.0210)	0.0001 (-0.0054 to 0.0057)
Re-admissions (30 days)	-0.0062 (-0.0277 to 0.0152)	0.0078 (-0.0034 to 0.0190)	**0.1805 (0.1624 to 0.1986)#**
**Secondary outcomes:**			
Total cost of 2° care services (£)~	14.4987 (-60.8355 to 89.8328)	23.1385 (-46.6572 to 92.9341)	83.3950 (-146.0357 to 312.8258)
Length of stay (days)	-0.0993 (-0.7057 to 0.5072)	0.2234 (-0.1919 to 0.6387)	**1.9649 (0.2253 to 3.7046)#**

**Outcome**	**Charlson Index**
	**Count (nbreg) model. Adjusted+ intervention effect (95% CI) (difference per patient per month)**
**Primary outcomes:**	
Inpatient non-electives	-0.0085 (-0.0227 to 0.0056)
Inpatient electives	0.0011 (-0.0197 to 0.0219)
Outpatient visits	-0.0340 (-0.1398 to 0.0718)
A&E visits	-0.0066 (-0.0303 to 0.0172)
ACSCs	**-0.0059 (-0.0099 to -0.0019)#**
Re-admissions (30 days)	**-0.0058 (-0.0106 to -0.0010)#**
**Secondary outcomes:**	
Total cost of 2° care services (£)~	-15.0060 (-73.2848 to 43.2728)
Length of stay (days)	0.0081 (-0.2412 to 0.2573)

+: adjusted for: age, imd domains (excluding health), practice- and time- fixed-effects. Marginal effects on *PICT x Post* reported.
*: standardised mean difference
#: significant at p<0.05
nbreg: negative binomial regression
~: zero-inflated negative binomial model based on admission events
n= 224,898 observations; 4098 individuals (period September 2010 to March 2015

References

1. Stagg, V., *CHARLSON: Stata module to calculate Charlson index of comorbidity.* Statistical Software Components, 2006.
2. HSCIC. *Quality and Outcomes Framework (QOF) - 2013-14*. 2014 25/08/2015]; Available from: http://www.hscic.gov.uk/catalogue/PUB15751.
3. Koller, D., et al., *Multimorbidity and long-term care dependency--a five-year follow-up.* BMC Geriatr, 2014. **14**: p. 70.
4. World Health Organization, *International statistical classification of diseases and health related problems (The) ICD-10*. 2004, World Health Organization.
